# Single Fraction and Hypofractionated Radiation Cause Cochlear Damage, Hearing Loss, and Reduced Viability of Merlin-Deficient Schwann Cells

**DOI:** 10.3390/cancers15102818

**Published:** 2023-05-18

**Authors:** Christine T. Dinh, Si Chen, Aida Nourbakhsh, Kyle Padgett, Perry Johnson, Stefania Goncalves, Olena Bracho, Esperanza Bas, Jorge Bohorquez, Paula V. Monje, Cristina Fernandez-Valle, Nagy Elsayyad, Xuezhong Liu, Scott M. Welford, Fred Telischi

**Affiliations:** 1 Department of Otolaryngology, University of Miami Miller School of Medicine, Miami, FL 33136, USAorb16@med.miami.edu (O.B.);; 2 Sylvester Comprehensive Cancer Center, University of Miami Miller School of Medicine, Miami, FL 33136, USA; 3 Department of Otolaryngology, University of Florida College of Medicine, Gainesville, FL 32610, USA; 4 Department of Radiation Oncology, University of Miami Miller School of Medicine, Miami, FL 33136, USA; 5 Department of Radiology, University of Miami Miller School of Medicine, Miami, FL 33136, USA; 6 Department of Radiation Oncology, University of Florida College of Medicine, Gainesville, FL 32610, USA; 7 Department of Research Pharmacy, Sylvester Comprehensive Cancer Center, Miami, FL 33136, USA; 8 Department of Biomedical Engineering, University of Miami, Miami, FL 33146, USA; 9 Department of Neurosurgery, University of Kentucky College of Medicine, Lexington, KY 40536, USA; 10 Burnett School of Biomedical Sciences, College of Medicine, University of Central Florida, Orlando, FL 32827, USA; cfv@ucf.edu; 11 Allina Health Cancer Institute—Radiation Oncology, St. Paul, MN 55102, USA; 12 Department of Human Genetics, University of Miami Miller School of Medicine, Miami, FL 33136, USA

**Keywords:** vestibular schwannoma, radiation, apoptosis, auditory hair cell, hearing, hearing loss, hypofractionation, cell death, Schwann

## Abstract

**Simple Summary:**

Vestibular schwannomas are benign tumors of the eighth cranial nerve, caused by mutations in the *NF2* gene encoding the tumor suppressor, merlin. A standard treatment for vestibular schwannoma is radiation therapy; however, radiation can cause sudden or progressive hearing loss in patients over time. Most commonly, vestibular schwannomas are treated with stereotactic radiation in one or multiple fractions. In this study, we investigated the effect of different radiation fractionation regimens on hearing in rats, loss of auditory hair cells, and control of tumor growth. Our work suggests that hypofractionation may improve the therapeutic ratio by increasing the likelihood of hearing preservation whilst equally reducing proliferation of tumor cells in comparison with single fraction. Further investigations are needed to understand radiation mechanisms and identify new therapeutic options to reduce hearing loss and enhance tumor control in vestibular schwannoma.

**Abstract:**

Background: Vestibular schwannomas (VS) are benign intracranial tumors caused by loss of function of the merlin tumor suppressor. We tested three hypotheses related to radiation, hearing loss (HL), and VS cell survival: (1) radiation causes HL by injuring auditory hair cells (AHC), (2) fractionation reduces radiation-induced HL, and (3) single fraction and equivalent appropriately dosed multi-fractions are equally effective at controlling VS growth. We investigated the effects of single fraction and hypofractionated radiation on hearing thresholds in rats, cell death pathways in rat cochleae, and viability of human merlin-deficient Schwann cells (MD-SC). Methods: Adult rats received cochlear irradiation with single fraction (0 to 18 Gray [Gy]) or hypofractionated radiation. Auditory brainstem response (ABR) testing was performed for 24 weeks. AHC viabilities were determined using immunohistochemistry. Neonatal rat cochleae were harvested after irradiation, and gene- and cell-based assays were conducted. MD-SCs were irradiated, and viability assays and immunofluorescence for DNA damage and cell cycle markers were performed. Results: Radiation caused dose-dependent and progressive HL in rats and AHC losses by promoting expression of apoptosis-associated genes and proteins. When compared to 12 Gy single fraction, hypofractionation caused smaller ABR threshold and pure tone average shifts and was more effective at reducing MD-SC viability. Conclusions: Investigations into the mechanisms of radiation ototoxicity and VS radiobiology will help determine optimal radiation regimens and identify potential therapies to mitigate radiation-induced HL and improve VS tumor control.

## 1. Introduction

Vestibular Schwannomas (VS), the most common tumor of the cerebellopontine angle and internal auditory canal, comprise 8–10% of all intracranial tumors, and is estimated to affect 1 in 1000 people during their lifetime [1]. They are benign intracranial tumors that originate from the Schwann cells of the vestibulocochlear nerve. They develop after biallelic inactivation of the *NF2* gene, which encodes a tumor suppressor called merlin. VS can cause hearing loss (HL), dizziness, and other neurological deficits due to compression of the brainstem and adjacent cranial nerves [2]. Management of VS includes observation with serial imaging, radiotherapy, and microsurgical resection, depending on the size of the tumor, the age of the patient, and clinical symptomatology. The main goal of radiotherapy in treating VS is to halt or reduce tumor growth, while minimizing radiation toxicity to adjacent neurovascular structures [3].

Stereotactic radiation is a form of external beam radiotherapy that provides a very sharp gradient or fall-off dose outside the target to limit scattered dose to surrounding normal tissue. When stereotactic radiation is given in one fraction, it is referred to as “stereotactic radiosurgery (SRS)”. When stereotactic radiation is given in multiple fractions, it is commonly referred to as “stereotactic radiotherapy (SRT)” or “fractionated stereotactic radiotherapy (FSRT)”. Because normal cells have a superior deoxyribonucleic acid (DNA) repair capacity than neoplastic cells, fractionation of radiotherapy can create a therapeutic window by leveraging the radiobiological differences between tumor and normal tissues, as with the cochlea [4]. Fractionation can also promote reoxygenation and cell cycle redistribution between fractions that sensitizes tumors to radiation [5]. Hypofractionation refers to the delivery of radiation with fractions > 2 Gray (Gy). For VS, common SRS protocols incorporate a marginal tumor dose of 11–14 Gy. Hypofractionated SRT protocols that have been employed for VS are 6 Gray for 3 fractions, 4 or 5 Gy for 5 fractions, and 3 Gy for 10 fraction, among others [6,7].

Using SRS, the long-term tumor control rates at 10 years for VS have been reported to be 88–91% [8,9]. However, 9–12% of VS will continue to progress over time after radiotherapy. Although the biological explanations for the following relationships are unclear, studies have shown that faster growing tumors (>2.5 mm/year in maximum diameter or volume doubling time >15 months), tumors associated with Neurofibromatosis Type 2 (NF2), and large tumors with volumes >6 cm^3^ are less responsive to SRS or SRT [10,11,12,13,14,15,16]. Furthermore, the failure rate may be underestimated since the rate of growth of VS may vary spontaneously over time, hence the lack of progression may spuriously increase the success rate of SRT [3,17].

Radiotherapy to the cerebellopontine angle or internal auditory canal inevitably exposes the cochlea and cochlear nerve to some dose of radiation. Several factors have been linked to radiation-induced HL in VS patients, including age, tumor size, pretreatment hearing, and cochlear irradiation dose [18,19,20]. Among these factors, cochlear dose is the only potentially modifiable factor in radiotherapy planning. Studies have shown that SRS dose (prescribed to the margin of the targeted tumor) of >13 Gy is associated with a higher risk of facial nerve injury and hearing loss [21,22]. Hence, commonly prescribed SRS doses are usually ≤13 Gy. However, even when limiting the marginal tumor dose to 12–13 Gy, long term studies showed that the rate of HL development is still very high, with up to 77% losing serviceable hearing at 10 years [19,23,24,25,26,27,28]. When the marginal tumor dose is reduced further to 11–12 Gy and/or the cochlear dose to <4 Gy, the rate of hearing preservation continues to remain low at approximately 50–57% at a median follow-up less than 5 years [29,30].

There is a paucity of literature suggesting that fractionation may mitigate radiation-induced HL by reducing toxicity to auditory hair cells (AHC) [31]. Although studies have shown good tumor control rates with FSRT, they have not demonstrated an advantage for preserving hearing in VS patients when compared to single fraction radiation [6,32,33,34,35,36]. A better understanding of the dose-response relationship and an elucidation of the molecular mechanisms involved in radiation-induced HL are important in identifying strategies to improve hearing preservation and tumor control rates. Thus, in this study, we aimed to: (1) develop an animal model to investigate the dose–response relationship between cochlear irradiation and HL, (2) understand potential molecular and cellular mechanisms involved in radiation injury to the cochlea, and (3) test the effect of single fraction versus hypofractionated radiation on the specificity and viability of normal Schwann cells (SC) and merlin-deficient Schwann cells (MD-SC; aka tumor cells).

## 2. Materials and Methods

### 2.1. Cell Cultures

Primary human Schwann cells (HSC), originally isolated from adult human peripheral nerve tissue, were obtained from the Monje Research Laboratory. These deidentified cells were expanded up to passage 2 and stored by cryopreservation using standard methods [37]. HSCs from 2 independent batches were selected for experimentation (designated as HSC1 and HSC2 in the figures). These cells were plated directly from a cryogenic stock and cultured at 37 °C and 5% CO_2_, on 0.01% poly-L-ornithine (PLO; Sigma, St. Louis, MO, USA) and laminin (5 µg/mL; Gibco, Life Technologies, Carlsbad, CA, USA)-coated flasks in mitogenic media for expansion of the stocks and/or direct experimentation, as previously described [37,38]. Mitogenic media was prepared with Dulbecco’s modified Eagle medium (DMEM; Gibco), 10% heat inactivated fetal bovine serum (FBS; Seradigm, VWR, Radnor, PA, USA), forskolin (2 μM; Sigma), heregulin (10 nM; Sigma), and 1% penicillin-streptomycin (Gibco). Human MD-SCs (HSC7228-45; aka MD-HSC-45 or HS01) and their isogenic normal SC control (HS11) were obtained from the Fernandez-Valle Research Laboratory and expanded on CellBIND plates (Corning, New York, NY, USA) using Schwann media (ScienCell, Carlsbad, CA, USA), as previously described [39]. All cells used in this study were established from cryogenic stocks from deidentified donors, and thus, experimentation with deidentified human cells constitutes non-human subjects research by the Human Subjects Research Office of the University of Miami.

### 2.2. Animals

All rats were treated in accordance with the Guide for the Care and Use of Laboratory Animals of the National Institutes of Health (8th Edition) [40]. The study was reviewed and approved by the University of Miami Internal Animal Care and Use Committee (Protocol #14-020). For audiometric studies, thirty-one adult male Norway Brown rats (250–300 g; Charles River Laboratories, Inc., Wilmington, MA, USA) were randomized to receive single-fraction (0, 3, 6, 9, 12, 15, or 18 Gy) or hypo-fractionated radiation (6 Gy daily × 3 or 4 Gy daily × 5) to the left ear and followed for 24 weeks. For mechanistic studies, thirty-six neonatal Sprague Dawley rats (Charles River Laboratories, Inc., Wilmington, MA, USA) were randomized to receive 0, 6, 12, or 18 Gy to both cochleae; cochleae were harvested at 6 h for real-time polymerase chain reaction (PCR) array and 72 h for cell death assays. Three additional adult Norway Brown rats and three neonatal Sprague Dawley rats were utilized for validation of cochlear irradiation regimens.

### 2.3. Custom Restrainer

Adult rats were placed in the prone position in a custom-made wooden platform restrainer. Rigid head fixation was accomplished by anchoring the upper incisors on an O-ring secured at one end of the restrainer. A soft foam spacer was placed on the right and left sides of the skull to prevent head movement. A prolene suture (Ethicon) anchored at the center of the O-ring (between both central incisors) was positioned over the vertex, equidistant between bilateral medial and lateral canthi, serving as a midline marker. Lead sheeting (~8 mm) was placed over the platform restrainer to shield the rat from unintentional radiation exposure. A square area (~2 × 2 cm) above the left auricle and lateral to midline was left exposed, allowing radiation to be delivered to the cochlea while minimizing unnecessary brainstem exposure.

Neonatal rats were placed in a prone position in a custom-made pie-shaped restrainer. Neonatal rat heads were positioned between two metal rods and head fixation was accomplished by applying a soft, rubber neck collar to prevent movement of the head. Lead sheeting was placed over the pie restrainer to shield the body of the rat from unnecessary radiation. Due to their small size, whole head irradiation was performed for cochlear irradiation studies.

### 2.4. Cochlear Irradiation Protocols

The RS 2000 Biological Irradiator (Rad Source Technologies, Suwannee, GA, USA) is equipped with a 0.3 mm Copper filter and delivers a cone-shaped radiation beam at 160 kV and 25 mA in the vertical plane, using a dose rate of ~5.5 Gy/min. Metal-oxide-semiconductor field-effect transistor (MOSFET) dosimeter probes (2.5 mm × 0.3 mm, Best Medical, Ottawa, ON, Canada) were calibrated to a standard ion chamber sensor (10X6-0.6 high dose rate chamber, Radcal, Inc., Monrovia, CA, USA) attached to an Accu-dose Meter Dose (Radcal, Inc.).

**Figure 1:**
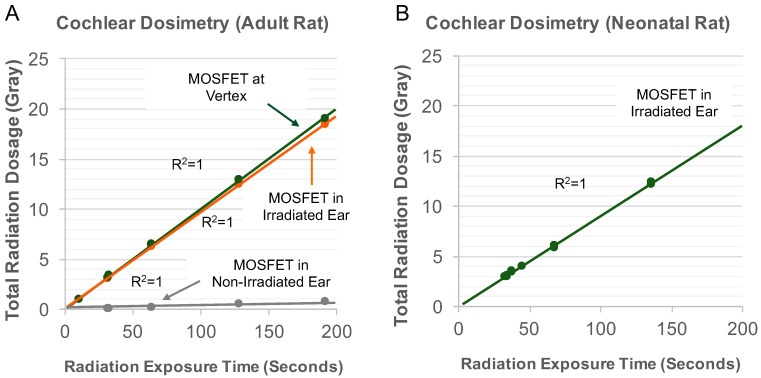
Validation of Adult and Neonatal Rat Irradiation Protocols. (**A**) In adult rats, there was a dose-dependent and linear relationship between the radiation exposure time (seconds) and the total radiation dosage received by metal oxide semiconductor field effect transistor (MOSFET) dosimeter probes at the vertex and irradiated ear (R^2^ = 1). The non-irradiated cochlea received negligible amounts of radiation. (**B**) In neonatal rats, there was also a dose-dependent and linear relationship between the radiation exposure time (seconds) and the total radiation dosage received by MOSFET dosimeter probes placed on bilateral cochlear promontories (R^2^ = 1). Circle marker = mean.

To validate adult rat cochlear irradiation protocols, bilateral wide-field myringotomy incisions were performed using binocular microscopy on three euthanized adult Norway Brown rats. Each rat phantom was placed in the custom restrainer and MOSFET probes were positioned on the cochlear promontory by passing the probe through both myringotomy incisions. The restrainer was placed into the irradiator and the vertex was measured 8 cm away from the irradiation source. Cochlear irradiation regimens were tested, and the actual dose of radiation received by the cochlea (0, 3, 4, 6, 9, 12, 15, and 18 Gy) was verified by measurements obtained from MOSFET probes (Figure 1A). Using validated irradiation protocols, adult rats were anesthetized with ketamine (40 mg/kg) and xylazine (5 mg/kg) mixture), placed in custom restrainers with appropriate lead sheeting, and randomized to receive single fraction and hypofractionated radiation. Radiation was delivered to the left cochlea at a dose rate of ~5.8 Gy/min (*n* = 3 or 4 per radiation condition).

To validate neonatal rat cochlear irradiation protocols, three neonatal rats were euthanized and placed in the pie restrainer. Retroauricular incisions were performed on the skulls of rats to expose the brainstem and both cochleae. Because the head of a neonatal rat is much smaller than an adult rat, one MOSFET probe was placed atop right and left cochleae for validation of cochlear radiation dosages. The restrainer was placed in the irradiator and the vertex was measured 11 cm away from the irradiation source. Like adult rats, neonatal cochlear radiation regimens (0, 6, 12, and 18 Gy) were validated using MOSFET probes (Figure 1B). Using validated protocols, unanesthetized neonatal rats were placed in custom restrainers with appropriate lead sheeting, and radiation was delivered to both cochleae at a dose rate of ~5.3 Gy/min.

Lastly, cultured normal SCs and MD-SCs were irradiated with single fraction radiation (0 or 12 Gy) and hypofractionated radiation (6 Gy daily × 3 days, or 4 Gy daily × 5 days) in a similar fashion.

### 2.5. Auditory Brainstem Response Testing

Auditory brainstem response (ABR) testing was performed on anesthetized rats prior to radiation and at 1, 4, 10, 16, and 24 weeks after irradiation, as previously described [41,42]. In brief, adult rats (*n* = 3–4 per radiation condition) were anesthetized with a mixture of intraperitoneal ketamine (40 mg/kg) and xylazine (5 mg/kg) and placed on an isothermal pad (Deltaphase) in a sound-proof double-walled audio booth. Subdermal electrodes were placed on the vertex (reference), both mastoids, and the left hind leg (ground). Using a commercial system (SmartEP and SmartEP-ASSR, Intelligent Hearing Systems, Miami, FL, USA) coupled to high frequency transducers and customized for high frequency ABR in rats, pure tone stimuli were delivered with insert earphones at an average stimulation rate of 78.13 stimuli/s (for 4, 8, and 16 kHz) and 158 stimuli/s (for 24 and 32 kHz). Appropriate masking was provided to the contralateral ear. For each frequency tested, ABR tests were performed in 10-decibel (dB) increments with at least 500 repetitions per dB HL (decibels hearing level) tested. Hearing thresholds were determined by identifying the lowest intensity that produced a recognizable ABR waveform and applying objective detection with T square statistics applied to Quasi Steady State Responses (Amplitude > 0.05 µV; *p* < 0.05). Routine ear examinations were performed to confirm the absence of tympanic membrane perforation and otitis media. Pure tone averages (PTA) were determined by averaging ABR hearing thresholds at 4, 8, 16, 24, and 32 kHz, and PTA shifts were calculated by subtracting the PTA at 24 weeks and at baseline.

### 2.6. Auditory Hair Cell Viability Counts

When adult rats completed their 24-week ABR tests, the rats were euthanized and bilateral temporal bones were harvested, fixed in 4% paraformaldehyde, and decalcified in 10% ethylenediaminetetraaceticacid (EDTA) for 3 weeks. The organs of Corti (OC) were dissected under a microscope, permeabilized with 1% Triton in phosphate buffered saline (PBS), and stained with fluorescein isothiocyanate (FITC)-labeled phalloidin (Sigma) to visualize the cuticular plates and stereocilia of AHCs. The specimens were then mounted on slides with anti-fade medium and cover slipped. Representative images of the apical, middle, and basal turns were obtained using a confocal microscope with a 40× oil immersion lens (Carl Zeiss LSM 700 Laser Scanning Confocal Microscope, Oberkochen, Germany). The number of viable inner hair cells (IHC) and outer hair cells (OHC) were counted over 150 µm representative segments, and the percentages of IHC and OHC losses were calculated for each turn of the cochlea (*n* = 3 per radiation condition). An AHC was considered viable if it possessed an intact stereociliary bundle and cuticular plate.

### 2.7. Immunohistochemistry and Immunofluorescence

One cochlea from the 0 Gy, 12 Gy, 6 Gy daily × 3, and 4 Gy daily × 5 groups were reserved for immunofluorescence and immunohistochemistry. After fixation and decalcification, cochleae were embedded in optimal cutting temperature medium, snap frozen in liquid nitrogen, and sectioned with a cryostat (CM1860, Leica Biosystems, Inc., Heidelberger, Germany). Ten-micrometer thick sections were mounted on positively charged glass slides and processed with hematoxylin and eosin (H&E) to visualize the morphology of the stria vascularis and spiral ligament using a light microscope (10× magnification). Additional sections were permeabilized with 1% Triton X-100 (Sigma) in PBS, blocked with normal donkey serum (Calbiochem, San Diego, CA, USA), and incubated at 4 °C overnight in 1:200 rabbit anti-rat beta-tubulin antibody (ab15568, Abcam, Waltham, MA, USA), a marker for neurons. The specimens were then washed and incubated with donkey anti-rabbit secondary antibody conjugated to Alexa-488 (1:200, Invitrogen, Waltham, MA, USA) for 2 h and 4′6′-diamidino-2-phenylindole dihydrochloride (DAPI, D8542, Sigma) for 15 min at room temperature. Subsequently, the neurosensory epitheliums were mounted on a glass slide with anti-fade medium and cover slipped. Representative images of the spiral ganglion neurons at the modiolus were obtained using a confocal microscope (40× oil immersion lens).

For normal SCs and MD-SC cultures, 10,000 cells per well were seeded on 16-well culture slides (Nunc, Thermo Fischer Scientific, Waltham, MA, USA), precoated in PLO and laminin (5 µg/mL) in their respective media. After 48 h, media was changed to maintenance media consisting of DMEM with 10% FBS and 1% penicillin-streptomycin and then irradiated as described above. After 2 and 6 h, cells were fixed with 4% paraformaldehyde and permeabilized and blocked with 0.25% Triton X-100, 5% normal donkey serum in in PBS. Immunofluorescence was performed for the following antibodies: mouse phospho-histone variant H2AX (Ser139) antibody (1:200, GT2311, Invitrogen) for 6 h or rabbit p21 antibody (1:100, MA5-14949, Invitrogen) for 2 h at 4 °C overnight in 0.125% Triton X-100, and 2.5% normal donkey serum. After washing, donkey anti-mouse and anti-rabbit secondary antibodies conjugated to Alexa-594 or Alexa-488 (1:200; Invitrogen) were used for 1 h at room temperature. Finally, after another wash, the DAPI nuclear stain was added for 15 min. The slides were imaged using the Lionheart FX microscope (BioTek, Winooski, VT, USA). ImageJ software (NIH, Bethesda, MD, USA) was used to calculate the nuclear intensity of p21 and number of nuclear foci for γ-H2AX (Ser139) per high power field (*n* = 6 replicates per condition).

### 2.8. Polymerase Chain Reaction for Cell Death Genes

To investigate the molecular mechanisms behind high dose radiation, OC explants (*n* = 6 per condition) were harvested from neonatal rats 6 h after cochlear irradiation (0, 6, 12, and 18 Gy). Total RNA isolation was performed using the RNeasy Mini Kit (Qiagen, Germantown, MD, USA) and converted to cDNA using the RT^2^ First Strand Kit (Qiagen). The expression of key cell death genes associated with necrosis and apoptosis were investigated using the Rat Necrosis RT^2^ Profiler PCR Array (#330231; Qiagen) and CFX96 Real-time PCR Detection System (Bio-rad, Hercules, CA, USA), per the manufacturer’s protocol. A list of all the genes and acronyms can be found in Supplemental Table S1. Data was normalized to housekeeping genes and analyzed using the RT^2^ Profiler PCR Array Data Analysis Portal (Version 3.5; Qiagen). The fold change was determined by calculating the ratio between threshold cycle (C_T_) of irradiated cochleae and non-irradiated cochleae (0 Gy). Mean fold changes >2-fold were confirmed using real-time polymerase chain reaction (PCR), primer pairs for Bax (R_Bax_1; KiCqStart, Sigma), Fas (R_Fas_1; KiCqStart, Sigma), and Pidd1 (R_Pidd_1; KiCqStart, Sigma), iQ SYBR Green Supermix (Bio-rad) and a CFX96 Real-time PCR Detection System (Bio-rad).

### 2.9. Cell Death Assay

To measure the cell death response in live AHCs, OC explants (*n* = 6 per condition) were harvested from neonatal rats at 72 h after cochlear irradiation (0, 6, 12, and 18 Gy) and placed in maintenance media consisting of DMEM, supplemented with glucose to 6 g/L, 1% N1 medium supplement (Sigma), and penicillin G (10 U/mL; Sigma). The apoptosis/necrosis detection kit (ab176750; Abcam) was utilized to simultaneously detect apoptosis, necrosis, and viable cells, per the manufacturer’s protocol. Phosphatidylserine (PS) expression is a hallmark and universal indicator of initial/intermediate stages of cell apoptosis. PS is expressed on the cell surface before morphological changes are observed and can be detected with the PS sensor (Excitation (Ex)/Emission (Em) = 630/660 nm), which emits a red fluorescence in the presence of PS. Loss of plasma membrane integrity can be detected with DNA Nuclear Green DCS1, which is a membrane-impermeable nuclear dye that emits a green fluorescence (Ex/Em = 490/525 nm). DCS1 labels nuclei in necrotic cells. CytoCalcein Violet 450 is a dye that accumulates in the cytoplasm of live cells and can be detected as blue fluorescence (Ex/Em = 405/450 nm). Subsequently, OC explants were mounted on a slide with anti-fade medium. Because the basal turn is most susceptible to radiation injury, representative images of the basal turn were obtained using a confocal microscope at 40× magnification with an oil immersion lens. The percentage of IHCs and OHCs over 150 µm segments expressing markers for apoptosis, necrosis, and viability were measured.

### 2.10. Live Cell Imaging and Viability Assays for Schwann Cells and Merlin-Deficient Schwann Cells

For cell-based assays, 10,000 cells were cultured on 96-well plates (precoated with 0.01% PLO and laminin (5 ug/mL)) in respective media at 37 °C and 5% CO_2_. After 48 h, the media was changed to maintenance media and exposed to single fraction and hypofractionated radiation regimens as described above. Live cell phase contrast light imaging (*n* = 3 wells per condition) was performed daily using the Lionheart FX microscope (BioTek) and associated Gen5 Software was used to measure cell counts over time. At 96 h post-irradiation, cell viability was measured using CellTiter-Glo (Promega, Madison, WI, USA) per manufacturer’s recommended instructions (*n* = 6 wells per condition). Luminescence in relative luminescence units (RLU) was measured with the Glomax luminometer (Promega), and mean fold change (relative to 0 Gy) was calculated.

### 2.11. Statistical Analysis

To validate our radiation protocols, linear regression analysis was utilized for measurements obtained from MOSFET dosimeter probes. One- and two-way analyses of variance with post hoc Tukey test was utilized to compare differences in ABR threshold shifts, PTA shifts, cell viability, gene expression data sets, and immunofluorescence counts. Mann Whitney U and Kruskal Wallis non-parametric tests were utilized when distributions were not normal. Significance was set at *p* < 0.05. Statistical analysis was performed using SAS 9.4.

## 3. Results

### 3.1. Validation of Cochlear Irradiation Protocols Using MOSFET Probes

In the adult rat protocol, various dosages of radiation were delivered to the left ear, and MOSFET probes were utilized to measure total radiation dosage received at the vertex, in the irradiated left ear, and in the non-irradiated right ear. There was a dose-dependent and linear relationship between the radiation exposure time (seconds) and the total radiation dosage received by the MOSFET dosimeter probes at the vertex (y = 0.1x; R^2^ = 0.99964) and irradiated ear (y = 0.0967x; R^2^ = 0.99991). In addition, there were negligible amounts of radiation delivered to the non-irradiated ear (y = 0.0038x; R^2^ = 0.99959), thus validating the irradiation protocol in adult rats (Figure 1A).

In the neonatal rat protocol, various dosages of radiation were delivered to both cochleae, and the total dosage of radiation received by MOSFET probes located on cochlear promontories were measured. Like adult rats, there was a dose-dependent and linear relationship between the radiation exposure time (seconds) and the total radiation dosage received by MOSFET dosimeters (y = 0.0906x − 0.078; R^2^ = 0.99910), thereby validating the irradiation protocol in neonatal rats (Figure 1B).

### 3.2. Auditory Brainstem Response Testing after Single Fraction Cochlear Irradiation

**Figure 2:**
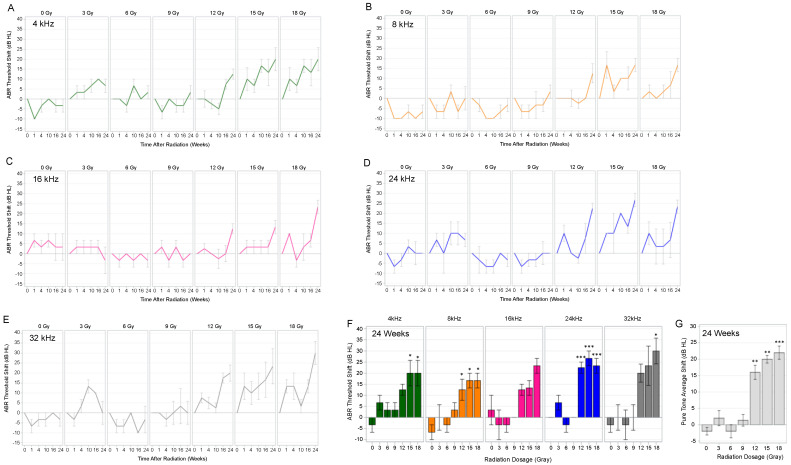
Auditory Brainstem Response (ABR) Threshold Shifts for Single Fraction Radiation. (**A**–**E**) ABR threshold shifts are depicted with line graphs for 4, 8, 16, 24, and 32 kHz frequencies from baseline to 24 weeks after cochlear irradiation. Cochlear radiation doses ≥12 Gy initiated progressive ABR threshold shifts at various frequencies, predominantly at 16 and 24 weeks. (**F**) Depending on the frequency, ears exposed to 12, 15, and 18 Gy had significantly higher ABR threshold shifts at the study endpoint (24 weeks) than those exposed to 0 Gy. (**G**) Shifts in pure tone average (PTA) were also significantly greater with cochlear radiation doses ≥12 Gy at 24 weeks. Line and bar correspond to mean. Error bars represent ± standard error mean. * *p* < 0.05. ** *p* < 0.01. *** *p* < 0.001. dB HL, decibels hearing level.

ABR thresholds at 4, 8, 16, 24, and 32 kHz were measured at baseline and at 1, 4, 10, 16, and 24 weeks after single fraction cochlear irradiation (0, 3, 6, 9, 12, 15, and 18 Gy). The ABR threshold shifts from baseline for the five tested frequencies are depicted in Figure 2A–E. In adult rats, shifts in ABR thresholds were more prevalent when cochleae received ≥12 Gy of radiation; the ABR threshold shifts were also progressive, occurring predominantly at the 16- and 24-week time points. By the study endpoint of 24 weeks, high dose radiation (≥12 Gy) initiated significant increases in ABR thresholds depending on the frequency when compared to baseline (Figure 2F). This was also evident when analyzing the PTA, which showed that cochlear irradiation doses ≥12 Gy initiated significant increases in PTA at 24 weeks (Figure 2G).

### 3.3. Auditory Hair Cell Viability Counts after Single Fraction Cochlear Irradiation

**Figure 3:**
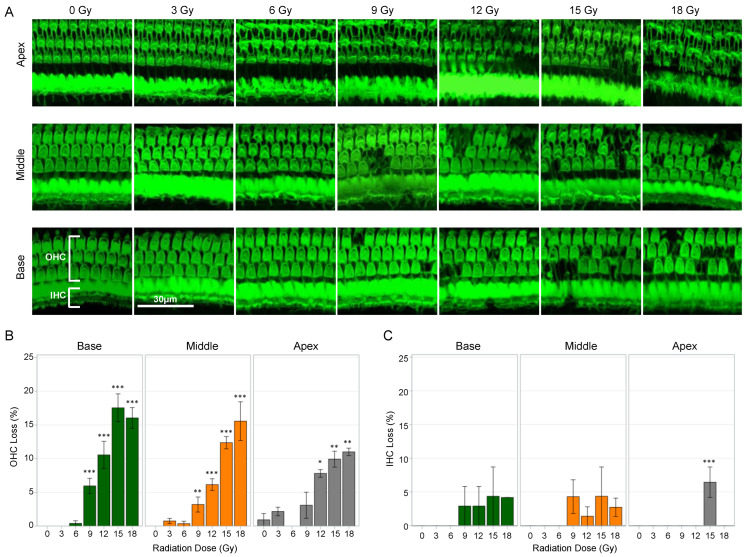
Auditory Hair Cell (AHC) Viability following Single Fraction Radiation. (**A**) Cochleae were harvested, fixed, and stained with fluorescein-isothiocyanate (green) at 24 weeks after single fraction irradiation (0, 3, 6, 9, 12, 15, or 18 Gy). Representative images of the apical, middle, and basal turns of the basilar membrane show three rows of outer hair cells (OHC) and one row of inner hair cells (IHC). Loss of AHCs was more evident in all turns of the cochleae at 9, 12, 15, and 18 Gy, when compared to 0, 3, and 6 Gy. With higher doses of cochlear irradiation, there was a preferential loss of OHCs over IHCs. (**B**) The percentages of OHC and IHC loss were calculated for all turns of cochlea. When compared to 0 Gy, there were significantly higher percentages of OHC loss at 9 (basal and middle turns) and 12–18 Gy conditions (all turns). There were no significant differences in OHC loss between the basal, middle, and apical turns within each radiation dose. (**C**) When compared to non-irradiated controls, there were no significant differences in the percentages of IHC loss in cochleae exposed to 3, 6, 9, 12, 15, and 18 Gy, except for 15 Gy at the apex. Bars represent mean percentage. Error bars correspond to ± standard error mean. * *p* < 0.05. ** *p* < 0.01. *** *p* < 0.001.

At 24 weeks after cochlear irradiation, cochleae were harvested and fixed, and representative FITC-stained images of the apical, middle, and basal turns were obtained (Figure 3A). Cochleae exposed to 0, 3, and 6 Gy of radiation had preservation of the three rows of outer hair cells (OHC) and one row of inner hair cells (IHC). Cochleae that were exposed to 9, 12, 15, and 18 Gy of radiation demonstrated OHC and IHC losses in all cochlear turns. The percentages of OHC and IHC losses were calculated for all turns of the cochleae (Figure 3B and Figure 3C, respectively). In cochleae exposed to ≥9 Gy of radiation, OHC losses were higher in the basal, middle, and apical turns of the cochleae, except for the apical turn of 9 Gy, where increases were not significant (Figure 3B). The basal turns experienced more OHC losses than the middle and apical turns; however, the differences did not meet statistical significance within each radiation dosage (*p* > 0.05). In addition, radiation preferentially affected OHC over IHCs. When compared to non-irradiated controls, there were no significant differences in IHC loss in all three cochlear turns and at all radiation dosages (*p* > 0.05), except for the apical turn for 15 Gy (*p* < 0.0001) (Figure 3C).

### 3.4. Cell-Based Assay for Apoptosis, Necrosis, and Viability after Single Fraction Cochlear Irradiation

**Figure 4:**
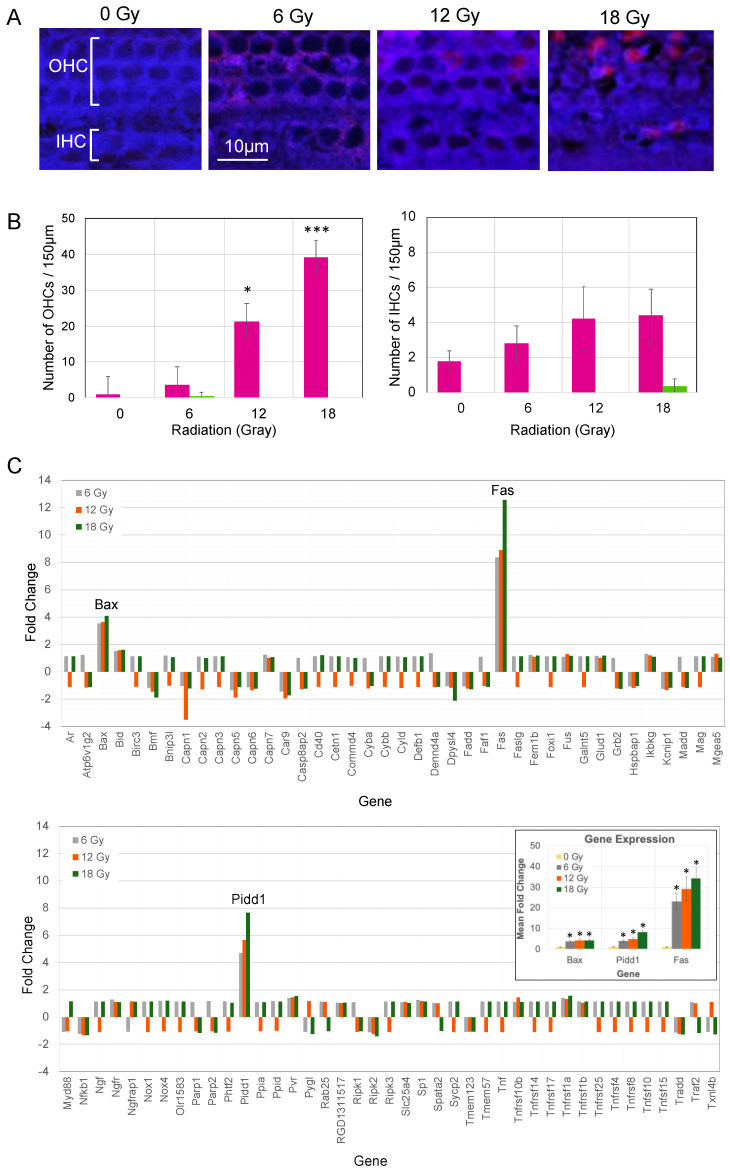
Apoptotic Cell Death of Auditory Hair Cells (AHC) following Single Fraction Radiation. Neonatal rat organs of Corti were harvested at 6 and 72 h following cochlear irradiation in vivo. (**A**) A cell death assay was performed ex vivo on organs of Corti harvested 72 h after cochlear irradiation. At 0, 6, and 12 Gy, most outer hair cells (OHC) and inner hair cells (IHC) demonstrated cytoplasmic uptake of cytocalcein violet 450 (blue), a marker of cell viability. At 12 and 18 Gy, OHCs and IHCs demonstrated cytoplasmic uptake of apoptosis marker, phosphytidylserine sensor (pink), compared to the 0 and 6 Gy conditions. There was minimal to no expression of DNA nuclear green stain DCS1 (green), a marker of necrosis. (**B**) There were significantly higher numbers of apoptotic OHCs per 150 µm lengths of the basal turn in cochleae exposed to 12 and 18 Gy when compared to 0 Gy controls. No differences were observed in the number of apoptotic IHCs between groups. The numbers of necrotic IHCs and OHCs were negligible. These findings together suggest that higher doses of radiation can initiate AHC death predominantly through apoptosis (not necrosis). Bars represent mean. Error bars correspond to ± standard error mean. * *p* < 0.05. *** *p* < 0.001. (**C**) A polymerase chain reaction (PCR) array was utilized to profile cell death genes from organs of Corti at 6 h post-irradiation. Using the PCR array, >2-fold up regulations of pro-apoptotic genes *Bax*, *Fas*, and *Pidd1* were demonstrated in cochleae exposed to 6, 12, and 18 Gy when compared to 0 Gy. These findings were confirmed with real-time PCR. Bars represent mean fold changes. Error bars correspond to ± standard error mean. * *p* < 0.001.

To determine whether auditory HCs were viable and/or undergoing apoptosis or necrosis, a cell death assay was performed ex vivo on organs of Corti harvested 72 h after cochlear irradiation (Figure 4A,B). In organs of Corti exposed to 0, 6, and 12 Gy, most OHCs and IHCs demonstrated cytoplasmic uptake of cytocalcein violet 450 (marker of cell viability, blue) around a nuclear shadow (Figure 4A). When compared to the 0 and 6 Gy conditions, OHCs and IHCs in cochleae exposed to 12 and 18 Gy had higher levels of the apoptotic PS sensor (pink enhancement on Figure 4A). Very few auditory HCs expressed the DCS1 stain for necrosis (green enhancement).

The number of OHCs and IHCs expressing the PS sensor of apoptosis and DCS1 stain for necrosis were counted over 150 µm lengths of the basal turn (Figure 4B). The average numbers of apoptotic OHCs per 150 µm were significantly higher in cochleae exposed to 12 and 18 Gy (*p* = 0.043 and *p* = 0.0002, respectively) when compared to 0 Gy. There were no significant differences in the average number of apoptotic OHCs between 0 and 6 Gy conditions (*p* = 0.989), nor the average number of apoptotic IHCs between 0, 6, 12, and 18 Gy groups (*p* = 0.480). Furthermore, few to no cells demonstrated green DCS1 staining (marker of necrosis).

### 3.5. PCR for Cell Death Genes after Single Fraction Cochlear Irradiation

The Rat Necrosis RT^2^ Profiler PCR Array was utilized to determine gene expression levels of apoptosis, necrosis, and cell survival genes in neonatal organs of Corti 6 h after cochlear irradiation. The fold change in gene expression levels after exposure to 6, 12, and 18 Gy of radiation were calculated relative to 0 Gy controls and displayed in Figure 4C. The expression levels of pro-apoptotic genes, *Bax, Fas,* and *Pidd1,* were >2-fold in organs of Corti exposed to 6, 12, and 18 Gy compared to non-irradiated controls (Figure 4C). Real-time PCR was performed to confirm expression levels of *Bax, Fas,* and *Pidd1.* At 6 h, there were significant up regulations of all three pro-apoptosis genes when organs of Corti were exposed to 6, 12, and 18 Gy of radiation (*p* < 0.001).

### 3.6. Auditory Brainstem Response Testing after Hypofractionated Cochlear Irradiation

**Figure 5:**
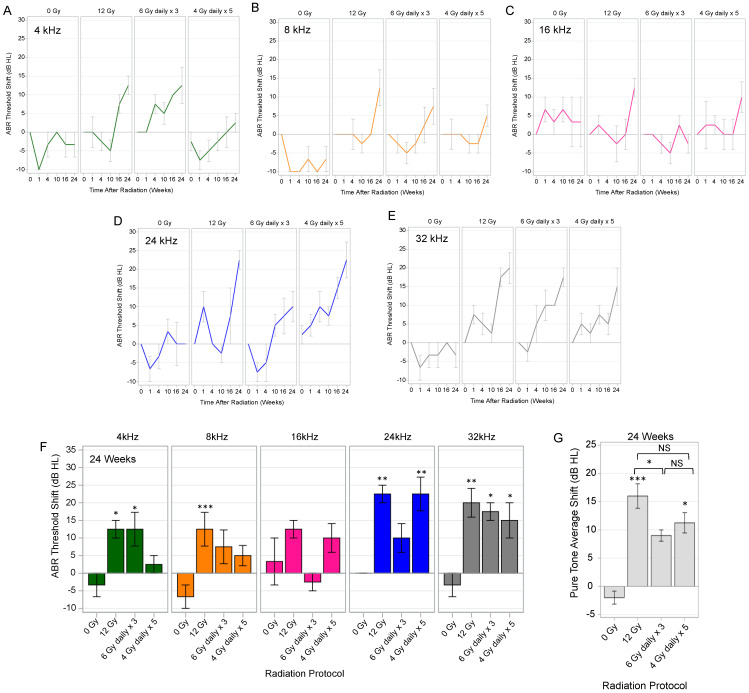
Auditory Brainstem Response (ABR) Threshold Shifts following Hypofractionated Radiation. (**A**–**E**) Adult rats received 0 Gy, 12 Gy, 6 Gy daily × 3, and 4 Gy daily × 5 to the left cochlea and ABR threshold shifts for 4, 8, 16, 24, and 32 kHz were measured from baseline to 24 weeks post-irradiation. ABR threshold shifts over time are depicted with line graphs for each frequency. (**F**) At 24 weeks post-irradiation, there were significant shifts in ABR thresholds in cochleae treated with 12 Gy, 6 Gy daily × 3, and 4 Gy daily × 5 when compared to non-irradiated controls at various frequencies, but predominantly in the 12 Gy condition. The ABR threshold shifts at 16 kHz were not significantly different from 0 Gy. (**G**) Cochlear irradiation caused significant shifts in pure tone averages (PTA) for 12 Gy and 4 Gy daily × 5 conditions. Although 6 Gy daily × 3 initiated mild increases in the PTA, shifts were not significantly different from 0 Gy, but significantly less than 12 Gy single fraction. Lines and bars represent mean decibel hearing level (dB HL). Error bars represent standard error mean. * *p* < 0.05. ** *p* < 0.01. *** *p* < 0.001. NS, not significant.

To assess the effects of hypofractionation on ABR threshold shifts (Figure 5), the left cochleae of adult rats were irradiated with hypofractionated regimens (6 Gy daily × 3, and 4 Gy daily × 5), and subsequently compared to the 12 Gy as a single fraction and the non-irradiated control condition (0 Gy). ABR thresholds shifts at 4, 8, 16, 24, and 32 kHz were measured at baseline and at 1, 4, 10, 16, and 24 weeks after completion of irradiation regimens and displayed in Figure 5A–E. The ABR threshold shifts and PTA shifts at the study endpoint of 24 weeks are displayed in Figure 5F and Figure 5G, respectively. Overall, the effect of radiation on the ABR threshold shifts at 24 weeks vary based on frequency tested and radiation regimen utilized (Figure 5F). When cochleae received 12 Gy, there were significant ABR threshold shifts at 24 weeks for 4, 8, 24, and 32 kHz. Cochleae receiving 6 Gy daily × 3 had significant ABR threshold shifts at 4 and 32 kHz, while cochleae receiving 4 Gy daily × 5 had ABR threshold shifts at 24 and 32 kHz. When comparing radiation conditions, we found that 12 Gy and the 4 Gy daily × 5 hypofractionated regimens caused significant increases in PTA, when compared to 0 Gy (*p* = 0.0009 and *p* = 0.0242, respectively) (Figure 5G). The PTA shifts at 24 weeks were not significantly different between 0 Gy and 6 Gy daily × 3 (*p* = 0.1061). Furthermore, the PTA shift at 24 weeks was significantly less for 6 Gy daily × 3, when compared to 12 Gy single fraction (*p* = 0.0384), but no different between the two hypofractionated regimens (*p* = 0.7720).

### 3.7. Auditory Hair Cell Viability Counts after Hypofractionated Cochlear Irradiation

**Figure 6:**
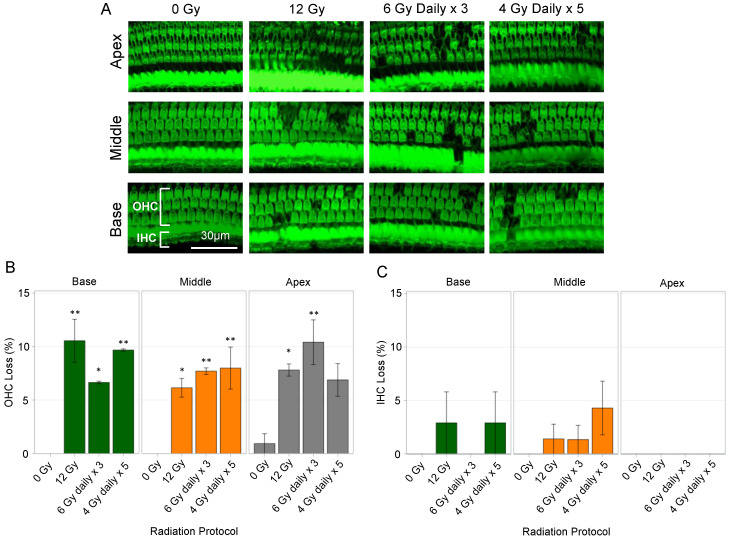
Auditory Hair Cell Viability following Hypofractionated Radiation. (**A**) Representative images of fluorescein isothiocyanate-stained organs of Corti demonstrate loss of outer hair cells (OHC) and inner hear cells (IHC) in cochleae irradiated with 12 Gy, 6 Gy daily × 3, and 4 Gy daily × 5 conditions. (**B**) There were higher percentages of OHC loss in cochleae receiving 12 Gy, 6 Gy daily × 3, and 4 Gy daily × 5 of radiation compared to non-irradiated cochleae at nearly all turns of the cochleae; however, there were no differences in percentage of OHC loss between the irradiated conditions. (**C**) Although IHC loss was identified in irradiated groups, the percentages of IHC loss were not different from non-irradiated cochleae. Bars represent mean values. Error bars correspond to ± standard error mean. * *p* < 0.05. ** *p* < 0.01.

Representative images of the apical, middle, and basal turns at 24 weeks show OHC loss in cochleae exposed to 12 Gy and hypofractionated regimens (6 Gy daily × 3, and 4 Gy daily × 5) (Figure 6A). The percentage of AHC losses were calculated in all three turns (Figure 6B). There were significantly higher percentages of OHC losses in the 12 Gy, 6 Gy daily × 3, and 4 Gy daily × 5 conditions when compared to 0 Gy, except for the apical turn in cochlea exposed to 4 Gy daily × 5; however, there were no differences in OHC loss between the three radiation conditions for the basal, middle, and apical turns (*p* > 0.05). With respect to percentages of IHC losses, there were no differences between all groups (*p* > 0.05).

### 3.8. Spiral Ganglion Neurons and Spiral Ligaments after Hypofractionated Cochlear Irradiation

**Figure 7:**
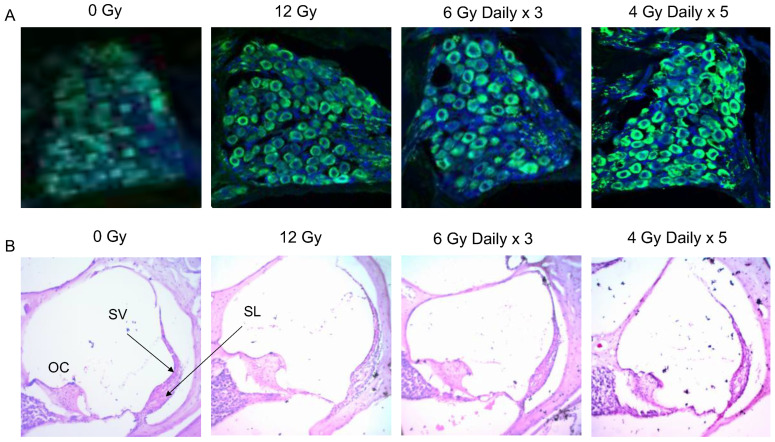
Immunohistochemistry of Spiral Ganglion Cell Bodies and Histology of the Spiral Ligament. Cochleae were harvested, fixed, decalcified, and sectioned 24 weeks after final radiation exposure. (**A**) Cross sections of the modiolus using a confocal microscope (40× oil immersion lens) demonstrate spiral ganglion cell bodies expressing beta-tubulin (green) and DAPI nuclear stain (blue). There were no obvious differences between the density and morphology of spiral ganglion cell bodies when cochleae were exposed to 0 Gy, 12 Gy, 6 Gy daily × 3, and 4 Gy daily × 5. (**B**) Cross sections of scala media were processed with hematoxylin and eosin staining to visualize the stria vascularis (SV) and spiral ligament (SL) and imaged using a light microscope (10× lens). There were no obvious differences in the cellular density and thickness of the SV between conditions. The spiral ligaments in cochleae exposed to 12 Gy and 4 Gy daily × 5 demonstrated hypocellularity compared to 0 Gy and 6 Gy daily × 3 conditions. These findings suggest that 12 Gy and 4 Gy daily × 5 of radiation may cause more severe alterations in cochlear homeostasis by affecting the cell density and function of the spiral ligament.

Histological sections of the spiral ganglion neuronal cell bodies were imaged with a confocal microscope after staining with DAPI nuclear stain (blue) and immunolabeling with beta-tubulin (green) (Figure 7A). There were no obvious differences in the density of spiral ganglion neuronal cell bodies between the 0 Gy non-irradiated cochlea and cochleae treated with 12 Gy single fraction and the 2 hypofractionated regimens (6 Gy daily × 3, and 4 Gy daily × 5). To determine the effect of radiation on the stria vascularis and spiral ligament, cross sections of the cochleae were stained with H&E (Figure 7B). No obvious differences in the cellular content or thickness of the stria vascularis were noticed; however, the spiral ligaments in the cochleae exposed to 12 Gy and 4 Gy daily × 5 regimens demonstrated noticeably less cellularity compared to 0 Gy and 6 Gy daily × 3 conditions.

### 3.9. Live Cell Imaging and Viability of Normal and Merlin-Deficient Schwann Cells after Hypofractionated Radiation

**Figure 8:**
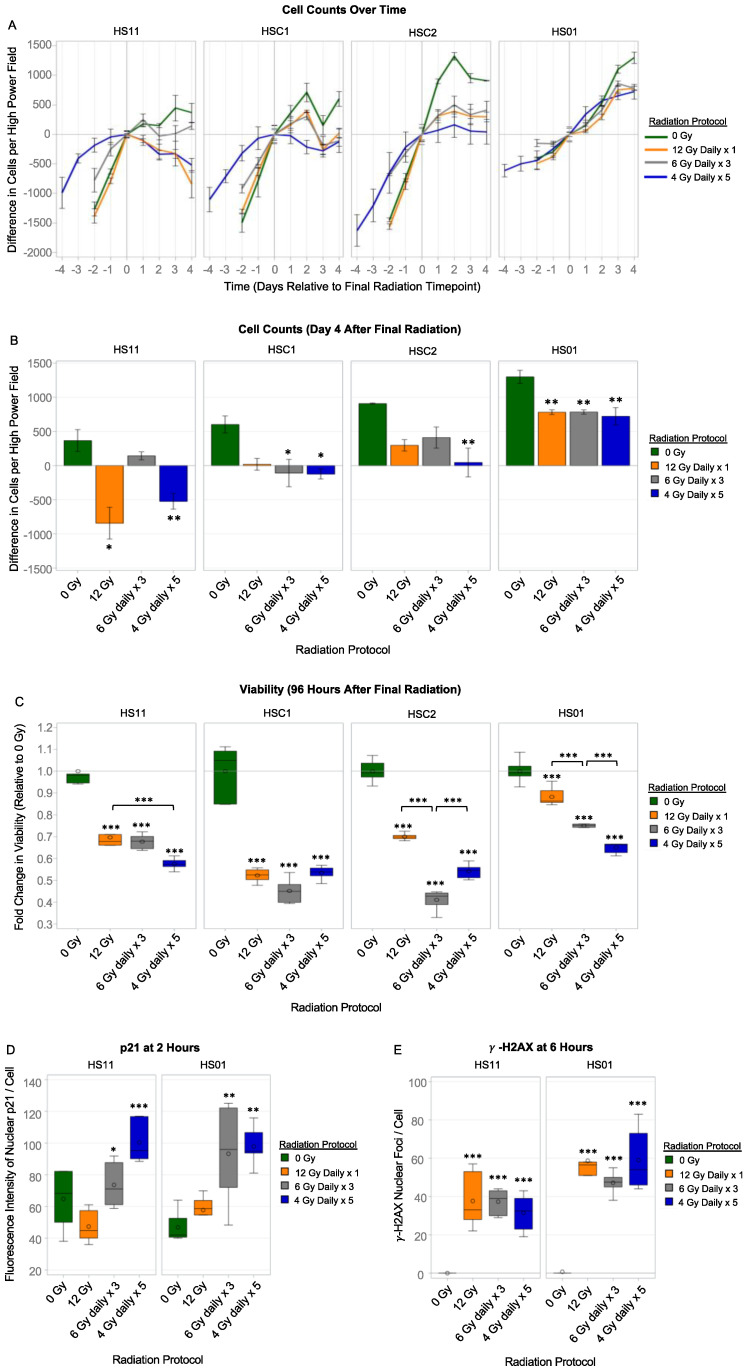
Viability, Cell Cycle Arrest, and DNA Damage in Irradiated Normal Schwann Cells (SC) and Merlin-Deficient Schwann Cells (MD-SCs). (**A**) Normal SCs (HS11, HSC1, HSC2) and MD-SCs (HS01) were exposed to 0 Gy, 12 Gy, 6 Gy daily × 3, and 4 Gy daily × 5 radiation regimens. Differences in cell counts over time (relative to final radiation timepoint) are depicted as line graphs. Overall, radiation caused reductions in viability in all four cell lines when compared to 0 Gy. Irradiated MD-SCs demonstrated delayed growth arrest when compared to normal SCs. (**B**) Viability assay at 96 h after final radiation dose. In accordance with cell counts, single-fraction and hypofractionated regimens caused reductions in viability in all cell lines when compared to 0 Gy. For MD-SCs (HS01), both hypofractionated regimens were more effective at reducing viability than 12 Gy single-fraction. (**C**) p21 Expression at 2 h after the final dose of radiation. There were significant upregulations of p21 in HS11 and HS01 cells after hypofractionated regimens when compared to both 0 and 12 Gy. There were no differences in p21 expression between 0 and 12 Gy single-fraction at 2 h. (**D**,**E**) Double-stranded DNA breaks, measured by the number of γ-H2AX nuclear foci per cell. In both HS11 and HS01 cells, single-fraction and hypofractionated radiation caused significant increases in γ-H2AX foci at 6 h after the final dose of radiation. Radiation initiated more γ-H2AX foci in HS01 cells compared to HS11, but this was not significant. Line and bars represent mean. Error bars correspond to ± standard error mean. Box plot with error bars represent minimum-25th/50th/75th percentiles-maximum. Circle marker = mean. * *p* < 0.05. ** *p* < 0.01. *** *p* < 0.001.

Normal SCs (HS11, HSC1 and HSC2) and MD-SCs (HS01) were irradiated with single fraction (0, 12 Gy) and hypofractionated (6 Gy daily × 3, and 4 Gy daily × 5) regimens and live cell phase contrast light imaging was performed daily. Initially, cell counts increased in a linear fashion in both the expanded, normal SCs, and MD-SCs; however, by the fourth day after final irradiation, all four cell cultures demonstrated obvious reductions in cell proliferation when compared to 0 Gy (Figure 8A,B). HS11 cells (isogenic control of HS01) appeared most susceptible to radiation-induced loss when compared to the other three cell cultures, particularly with 12 Gy and 4 Gy daily × 5. In general, the increases in viability over time observed in HSC1 and HSC2 cells plateaued or decreased early after exposure to all three radiation conditions. In contrast, increases in viability observed in MD-SCs (HS01) treated with 12 Gy and the 2 hypofractionated regimens demonstrated a plateau in a delayed fashion, i.e., on the fourth day after final radiation dose (Figure 8A); however, there were no significant differences in HS01 cell counts between the three radiation regimens (Figure 8B).

Viability was also measured at 96 h after the final dose of radiation using CellTiterGlo assay. Normal SCs (HS11, HSC1, and HSC2) and MD-SCs (HS01) demonstrated significant losses in viability after radiation with 12 Gy and both hypofractionated regimens (Figure 8C). The three normal SC cultures demonstrated larger reductions in viability after exposure to the three radiation regimens, than MD-SCs (HS01). For HS01, 6 Gy daily × 3 initiated significantly more loss in viability when compared to 12 Gy single fraction (*p* < 0.0001), and 4 Gy daily × 5 initiated significantly more loss in viability when compared to 6 Gy daily × 3 (*p* < 0.0001). Overall, these results are in accordance with the cell counts obtained from live cell imaging.

### 3.10. p21 and γ-H2AX Expression in Normal and Merlin-Deficient Schwann Cells after Hypofractionated Radiation

To detect levels of cell cycle arrest and radiation-induced double-strand DNA breaks, immunocytochemistry was performed at 2 h and 6 h for p21 and γ-H2AX, respectively. Both hypofractionated regimens initiated significant up regulation of nuclear p21 at 2 h in normal SCs (HS11) and MD-SCs (HS01) when compared to 0 Gy and 12 Gy single fraction (Figure 8C). When compared to 0 Gy, all three radiation regimens caused significantly high levels of DNA damage in HS11 and HS01 cells, as measured by the number of γ-H2AX foci. Overall, MD-SCs (HS01) developed higher levels of double-stranded DNA breaks when compared to normal isogenic Schwann cells (HS11), but this was not significant (*p* > 0.05) (Figure 8D).

## 4. Discussion

Ionizing radiation causes double-stranded DNA breaks that can initiate cell death in proliferating cells [43]. Because AHCs are post-mitotic cells of the neurosensory epithelium, it is believed that the cochlea is less susceptible to radiation injury [44]. Several studies have demonstrated high rates of unserviceable HL long-term after SRT. Some argue that this HL may be because of the VS microenvironment on the adjacent cochlea and cochlear nerve, rather than toxicity from radiation treatment [19,23,24,25,26,45,46]. However, retrospective studies assessing hearing preservation following SRT demonstrate significant variability, making it difficult to arrive at definitive conclusions from the available data. Such studies are heterogeneous regarding the choice of radiation regimen, cochlear radiation dose, tumor size, tumor location, tumor distance from the cochlea, baseline hearing, time of last follow-up, and other patient-associated factors, which likely play a role in determining the impact of radiation on HL in patients with VS and other skull base pathology. Thus, the contribution of radiation injury to HL in VS patients is subject to controversy.

In a systematic review of 11 retrospective investigations assessing the effect of radiation on hearing in VS patients, analysis of the results demonstrated a significant correlation between higher cochlear dose and worse hearing outcomes. However, there was considerable variation in the dosing parameters to the cochlea and the methods used to measure hearing outcomes. Due to this variability, the authors could not conclude that cochlear radiation dosing was an independent predictor of HL after SRT [47]. Although there are no prospective randomized clinical trials to assess the relationship between cochlear dose and hearing, most institutions aim for a cochlear mean dose < 4 Gy or a maximum dose of <9 Gy to minimize radiation-induced HL. For radiation regimens using three or five fractions, common metrics utilized are maximum cochlear doses < 17 Gy and <25 Gy, respectively.

In this study, we developed and validated a method to deliver precise radiation dosages to the cochlea in an animal model. We randomized adult rats to receive single fraction radiation (0, 3, 6, 9, 12, 15, and 18 Gy) to the left cochlea to evaluate the impact of radiation dose on hearing in a controlled setting, in the absence of VS. We found that higher doses of single fraction radiation caused progressive ABR threshold shifts over 24 weeks at multiple frequencies, and PTA shifts at 24 weeks were significant for 12, 15, and 18 Gy of cochlear irradiation. These findings generated a dose-response for single fraction radiation on HL in an adult rat model of cochlear irradiation. Furthermore, we assessed the viability of AHCs and found that radiation caused OHC losses with doses ≥9 Gy, with significant OHC losses observed in all cochlear turns when doses ≥12 Gy are used. Not only did we show that higher doses of radiation (≥12 Gy) affected all turns of the cochlea (basal, middle, and apical), but we also show that OHCs were more susceptible to radiation-induced loss than IHCs. Overall, these histologic findings are in accordance with patterns identified with ABR testing.

To assess the molecular mechanisms associated with radiation effect on hearing, we delivered cochlear irradiation (0, 6, 12, 18 Gy) to neonatal rats and harvested organs of Corti for gene expression profiling at 6 h and cell death assays at 72 h. We found that radiation at 12 and 18 Gy caused significant increases in the number of OHCs expressing cytoplasmic phosphytidylserine (apoptosis marker) when compared to 0 Gy. There was minimal to no expression of DCS1 (necrosis marker) in IHCs and OHCs in all conditions. Furthermore, gene expression analysis demonstrated >2-fold up regulations of pro-apoptotic genes *Bax*, *Fas*, and *Pidd1* in organs of Corti exposed to 6, 12, and 18 Gy, when compared to 0 Gy. Taken together with the in vivo ABR tests in adult rats, these findings indicate that higher doses of single fraction radiation cause progressive HL by initiating dose-dependent losses of OHCs through an apoptosis-dependent mechanism.

A common radiation regimen for the treatment of VS patients with serviceable hearing is a marginal tumor dose of 12 Gy; however, long term studies show high rates of developing unserviceable hearing, with rates up to 77% at 10 years [19,23,24,25,26]. In a retrospective investigation of 16 patients with meningiomas that extended into the internal auditory canal that received a median marginal tumor dose of 15 Gy, the actuarial incidence of HL at 5 years was 58%, suggesting that SRS can lead to cochlear toxicity in pathologies other than VS [48]. Thus, clinicians have utilized hypofractionated radiation or conventional fractionation in efforts to reduce the risk of radiation-induced HL while maintaining tumor control. A systematic review consisting of retrospective investigations and two quality of life prospective studies assessing single fraction and fractionated regimens (hypofractionation and conventional fractionation) for VS did not show a difference in hearing preservation among the groups [6]. To determine whether there is a hearing preservation advantage to using hypofractionated radiation, we tested common hypofractionated radiation regimens (6 Gy daily × 3 days, and 4 Gy daily × 5 days) against 12 Gy single fraction in our animal model. The hypofractionated radiation regimens tested in this study have been utilized in clinical practice for VS and are comparable when using linear-quadratic-based conversions for a tumor control probability of 90% in VS [6,7]. Overall, all three radiation regimens initiated progressive HL, when compared to 0 Gy. However, 12 Gy single fraction caused significant ABR threshold shifts in four frequencies (4, 8, 24, and 32 kHz), while both hypofractionated regimens initiated significant ABR threshold shifts in only two frequencies. When analyzing the PTA, we found that 12 Gy and 4 Gy daily × 5 caused significant shifts in PTA at 24 weeks, when compared to 0 Gy. Although 6 Gy daily × 3 caused an increase in the PTA, it was not significantly different than the 0 Gy control. More importantly, 6 Gy daily × 3 initiated significantly lower PTA shifts than 12 Gy single fraction, suggesting that 6 Gy daily × 3 may offer a potential hearing advantage when compared to 12 Gy single fraction.

On cochlear histology, 12 Gy single fraction and the two hypofractionated radiation regimens initiated significant OHC losses in all turns of the cochlea, except for the apical turn in the 4 Gy daily × 5 condition, where increases did not meet statistical significance. However, there were no differences between the percentages of OHC loss between the three radiation regimens. To understand how these radiation regimens can affect other parts of the cochlea, we performed a qualitative assessment of the spiral ganglion neuronal bodies as well as the stria vascularis and spiral ligament. We found that 12 Gy and the two hypofractionated radiation regimens did not affect the density and morphology of spiral ganglion cell bodies in irradiated cochlea, compared to 0 Gy, nor did they appear to alter the cell density and thickness of the stria vascularis. However, spiral ligaments exposed to 12 Gy and the 4 Gy daily × 5 conditions demonstrated less cellularity than cochlea exposed to 6 Gy daily × 3 or the 0 Gy conditions, indicating that 6 Gy daily × 3 may be more beneficial at preserving the spiral ligament important for maintaining homeostasis of the cochlea.

It is difficult to determine the best radiation regimen that preserves hearing without addressing how that radiation regimen may affect tumor control in VS. Overall, studies have shown that SRT has good control rates in VS. In a systematic review of 55 investigations, there were 6 non-randomized studies assessing tumor control rates between single fraction and fractionated regimens. The 5-year tumor control rates pooled from these studies showed no statistically significant difference in tumor control rate between cohorts [6]. However, the results of this study are limited by the retrospective nature of the non-randomized studies. To address the effectiveness of radiation on reducing the viability of tumor cells, we irradiated normal SCs and MD-SCs with single fraction and hypofractionated regimens used in our in vivo studies. We found that all three radiation regimens caused significant DNA damage and reductions in viability of normal SCs and MD-SCs, when compared to 0 Gy. Even though normal SCs were susceptible to radiation-induced injury in vitro, we do not expect normal SCs to express this degree of injury in vivo as SCs in their differentiated state do not proliferate under normal conditions and irradiated spiral ganglion neuronal bodies remained intact (Figure 7A). Both hypofractionated regimens initiated an upregulation of p21 at 2 h, suggesting that hypofractionation can cause sufficient radiation-induced DNA damage that can activate cell cycle arrest, earlier than that seen with 12 Gy single fraction. Furthermore, both hypofractionated regimens were more effective at reducing viability of MD-SCs than 12 Gy single fraction, with 4 Gy daily × 5 being more effective than 6 Gy daily × 3 at the 96-h time point tested. This finding is important because we showed that 6 Gy daily × 3 may have a hearing advantage over the other 2 radiation regimens but may not be as effective as 4 Gy daily × 5 in controlling proliferation of MD-SCs.

The limitations of our study are inherent in our in vitro and in vivo experiments, where findings may not accurately reflect what occurs in patients with VS. Although we evaluated hearing thresholds and cochlear histology in irradiated animals, we cannot test the effect of radiation on speech discrimination in rats, and animals did not harbor VS tumors. Future investigations would need to use genetically engineered or xenograft models of VS [49,50]. Furthermore, the results of this limited dose-response investigation may not reflect what is observed in the population at large where there is variability in tumor characteristics and baseline hearing level. Although hearing in animals was assessed for 24 weeks, more significant radiation changes to hearing thresholds and cochlear histology may occur at later time points. In our study, we assessed single fraction radiation and two hypofractionated regimens, other hypofractionated and conventional fractionated regimens were not assessed. Finally, our merlin deficient cell lines that originate from humans may not represent the full spectrum of heterogeneity seen in VS tumors, and future investigations should also involve the assessment of radiation on primary VS cells in vitro and in vivo, on apoptosis signaling pathways, and DNA repair mechanisms.

## 5. Conclusions

In conclusion, we have developed and validated an animal model to investigate the effects of radiation on hearing preservation. With our model, we were able to demonstrate that the cochlea is sensitive to radiation in a dose-dependent manner, show that OHCs are susceptible to radiation-induced cell death through apoptosis-related mechanisms, and provide a dose-response relationship using several quantifiable endpoints.

In addition, our findings suggest that hypofractionation, as opposed to single fraction, may be more protective against radiation-induced HL and more effective at reducing viability in MD-SCs than 12 Gy as a single fraction. Further investigations into radiation ototoxicity and the radiobiology of VS tumors can help us optimize radiation regimens or identify target-specific therapies that may improve treatment outcomes in VS patients.

## Data Availability

The data that support the findings of this study are available from the corresponding author upon reasonable request.

## Supplementary Materials

The following supporting information can be downloaded at: https://www.mdpi.com/article/10.3390/cancers15102818/s1, Supplemental Table S1. Names of Genes in RT² Profiler PCR Array Rat Necrosis (PARN-141ZD; Qiagen).
