# Supplementary material for: Single Fraction and Hypofractionated Radiation Cause Cochlear Damage, Hearing Loss, and Reduced Viability of Merlin-Deficient Schwann Cells
Source: Cancers (Basel). 2023 May 18;15(10):2818. doi: 10.3390/cancers15102818 (PMC10216287; doi:10.3390/cancers15102818)
Supplement: Supplementary file 1 [file cancers-15-02818-s001.zip › cancers-2325313-supplementary.pdf]

Supplemental Table S1. Names of Genes in RT² Profiler PCR Array Rat Necrosis (PARN-141ZD; Qiagen)

Acronym	Name
Aifm1	Apoptosis-inducing factor, mitochondrion-associated 1
Ar	Androgen receptor
Atp6v1g2	ATPase, H+ transporting, lysosomal V1 subunit G2
Bax	Bcl2-associated X protein
Bid	BH3 interacting domain death agonist
Birc3	Baculoviral IAP repeat-containing 3
Bmf	Bcl2 modifying factor
Bnip3l	BCL2/adenovirus E1B interacting protein 3-like
Capn1	Calpain 1
Capn2	Calpain 2
Capn3	Calpain 3
Capn5	Calpain 5
Capn6	Calpain 6
Capn7	Calpain 7
Capns1	Calpain, small subunit 1
Car9	Carbonic anhydrase 9
Casp8ap2	Caspase 8 associated protein 2
Cd40	CD40 molecule, TNF receptor superfamily member 5
Cetn1	Centrin, EF-hand protein, 1
Commd4	COMM domain containing 4
Cyba	Cytochrome b-245, alpha polypeptide
Cybb	Cytochrome b-245, beta polypeptide
Cyld	Cylindromatosis (turban tumor syndrome)
Defb1	Defensin beta 1
Dpysl4	Dihydropyrimidinase-like 4
Fadd	Fas (TNFRSF6)-associated via death domain
Faf1	Fas (TNFRSF6) associated factor 1
Fas	Fas (TNF receptor superfamily, member 6)
Faslg	Fas ligand (TNF superfamily, member 6)
Fem1b	Fem-1 homolog b (C. elegans)
Foxi1	Forkhead box I1
Fus	Fusion (involved in t(12;16) in malignant liposarcoma) (human)
Galnt5	UDP-N-acetyl-alpha-D-galactosamine:polypeptide N-acetylgalactosaminyltransferase 5 (GalNAc-T5)
Glud1	Glutamate dehydrogenase 1
Glul	Glutamate-ammonia ligase (glutamine synthetase)
Grb2	Growth factor receptor bound protein 2
Hspbap1	Hspb associated protein 1
Ikbkg	Inhibitor of kappa light polypeptide gene enhancer in B-cells, kinase gamma
Kcnip1	Kv channel-interacting protein 1
Lrdd	Leucine-rich repeats and death domain containing
Madd	MAP-kinase activating death domain
Mag	Myelin-associated glycoprotein
Mgea5	Meningioma expressed antigen 5 (hyaluronidase)

Myd88	Myeloid differentiation primary response gene 88
Symbol	Description
Nfkb1	Nuclear factor of kappa light polypeptide gene enhancer in B-cells 1
Ngf	Nerve growth factor (beta polypeptide)
Ngfr	Nerve growth factor receptor (TNFR superfamily, member 16)
Ngfrap1	Nerve growth factor receptor (TNFRSF16) associated protein 1
Nox1	NADPH oxidase 1
Nox4	NADPH oxidase 4
Olr1583	Olfactory receptor 1583
Parp1	Poly (ADP-ribose) polymerase 1
Parp2	Poly (ADP-ribose) polymerase 2
Phtf2	Putative homeodomain transcription factor 2
Ppia	Peptidylprolyl isomerase A (cyclophilin A)
Ppid	Peptidylprolyl isomerase D (cyclophilin D)
Pvr	Poliovirus receptor
Pygl	Phosphorylase, glycogen, liver
Rab25	RAB25, member RAS oncogene family
RGD1311517	Similar to RIKEN cDNA 9430015G10
RGD1562639	Similar to c-myc promoter binding protein
Ripk1	Receptor (TNFRSF)-interacting serine-threonine kinase 1
Ripk2	Receptor-interacting serine-threonine kinase 2
Ripk3	Receptor-interacting serine-threonine kinase 3
Slc25a4	Solute carrier family 25 (mitochondrial carrier; adenine nucleotide translocator), member 4
Sp1	Sp1 transcription factor
Spata2	Spermatogenesis associated 2
Sycp2	Synaptonemal complex protein 2
Tmem123	Transmembrane protein 123
Tmem57	Transmembrane protein 57
Tnf	Tumor necrosis factor (TNF superfamily, member 2)
Tnfrsf10b	Tumor necrosis factor receptor superfamily, member 10b
Tnfrsf14	Tumor necrosis factor receptor superfamily, member 14 (herpesvirus entry mediator)
Tnfrsf17	Tumor necrosis factor receptor superfamily, member 17
Tnfrsf1a	Tumor necrosis factor receptor superfamily, member 1a
Tnfrsf1b	Tumor necrosis factor receptor superfamily, member 1b
Tnfrsf25	Tumor necrosis factor receptor superfamily, member 25
Tnfrsf4	Tumor necrosis factor receptor superfamily, member 4
Tnfrsf8	Tumor necrosis factor receptor superfamily, member 8
Tnfsf10	Tumor necrosis factor (ligand) superfamily, member 10
Tnfsf15	Tumor necrosis factor (ligand) superfamily, member 15
Tradd	TNFRSF1A-associated via death domain
Traf2	Tnf receptor-associated factor 2
Txnl4b	Thioredoxin-like 4B
Actb	Actin, beta
B2m	Beta-2 microglobulin
Hprt1	Hypoxanthine phosphoribosyltransferase 1
Ldha	Lactate dehydrogenase A

Rplp1	Ribosomal protein, large, P1
RGDC	Rat Genomic DNA Contamination
